# Separate Developmental Programs for HLA-A and -B Cell Surface Expression during Differentiation from Embryonic Stem Cells to Lymphocytes, Adipocytes and Osteoblasts

**DOI:** 10.1371/journal.pone.0054366

**Published:** 2013-01-18

**Authors:** Hardee J. Sabir, Jan O. Nehlin, Diyako Qanie, Linda Harkness, Tatyana A. Prokhorova, Blagoy Blagoev, Moustapha Kassem, Adiba Isa, Torben Barington

**Affiliations:** 1 Department of Clinical Immunology, Odense University Hospital and Clinical Institute, University of Southern Denmark, Odense, Denmark; 2 Molecular Endocrinology Laboratory (KMEB), Department of Endocrinology, Odense University Hospital and University of Southern Denmark, Odense, Denmark; 3 Center for Experimental BioInformatics, Department of Biochemistry and Molecular Biology, University of Southern Denmark, Odense, Denmark; 4 Stem Cell Unit, Department of Anatomy, College of Medicine, King Saud University, Riyadh, Kingdom of Saudi Arabia; University of Kansas Medical Center, United States of America

## Abstract

A major problem of allogeneic stem cell therapy is immunologically mediated graft rejection. HLA class I A, B, and Cw antigens are crucial factors, but little is known of their respective expression on stem cells and their progenies. We have recently shown that locus-specific expression (HLA-A, but not -B) is seen on some multipotent stem cells, and this raises the question how this is in other stem cells and how it changes during differentiation. In this study, we have used flow cytometry to investigate the cell surface expression of HLA-A and -B on human embryonic stem cells (hESC), human hematopoietic stem cells (hHSC), human mesenchymal stem cells (hMSC) and their fully-differentiated progenies such as lymphocytes, adipocytes and osteoblasts. hESC showed extremely low levels of HLA-A and no -B. In contrast, multipotent hMSC and hHSC generally expressed higher levels of HLA-A and clearly HLA-B though at lower levels. IFNγ induced HLA-A to very high levels on both hESC and hMSC and HLA-B on hMSC. Even on hESC, a low expression of HLA-B was achieved. Differentiation of hMSC to osteoblasts downregulated HLA-A expression (P = 0.017). Interestingly HLA class I on T lymphocytes differed between different compartments. Mature bone marrow CD4^+^ and CD8^+^ T cells expressed similar HLA-A and -B levels as hHSC, while in the peripheral blood they expressed significantly more HLA-B7 (P = 0.0007 and P = 0.004 for CD4^+^ and CD8^+^ T cells, respectively). Thus different HLA loci are differentially regulated during differentiation of stem cells.

## Introduction

HLA class I molecules present cytoplasmic peptides to T-cell receptors on CD8^+^ T cells, which play a central role in the protection against viral and other intracellular infections as well as in immune reactions to neoplasms. Furthermore, certain HLA class I molecules play important roles as ligands for inhibitory NK-cell receptors. The presence or absence of HLA class I expression and its mode of regulation in various tissues are therefore of great importance for our understanding of T-cell and NK-cell mediated protection. In contrast to statements found in many authoritative text books of immunology claiming that HLA class I is expressed by all nucleated cells in the body [1]–[3], the expression is in fact lacking in several cell types [4]–[14]. Thus HLA class I expression is repeatedly reported as negative *in vivo* in neuronal cells of the brain, sperm and ova, placenta and islets of Langerhans [5]–[7], [9], [13], [15]. In fact, unequivocal evidence for cell surface HLA class I expression is limited to most cells in lymphoid tissues, epithelial cells of different body surfaces and the endothelial lining of blood vessels (excluding large vessels) [6], [7], [9], [10], [13], [14], [16]–[25]. Apart from these tissues, constitutive HLA class I expression is a matter of controversy. Skeletal muscle cells have been reported to express low amounts of HLA class I [6], [13] while other studies have found them to be negative [9], [11], [14]. Other examples are smooth muscle cells [6], [9], [13], [14], [25], [26], the parenchymatous cells of the thyroid and the adrenal glands [6], [9], [13], [27] and the kidney [8], [12] for which conflicting evidence has been reported. The discrepancies may be due to differences of specificity and sensitivity of the techniques used, because in most of the studies immunohistochemistry (IHC) was used where the read out is at best semi quantitative and different thresholds for positivity may be applied. In addition, it is difficult to compare the staining intensity between samples in different studies because different reagents and techniques were used. Class-specific or allele-specific HLA antibodies were developed originally for complement-dependent cytotoxicity assays (CDC) and flow cytometry. Establishing the sensitivity of such antibodies in IHC assays requires careful examination and validation which is not always undertaken.

Most studies that have addressed HLA class I expression in tissues used antibodies that detect HLA class I in general, most commonly the W6/32 or PA2.6 monoclonal antibodies. W6/32 is well known for binding to all HLA class I alleles [5]. It is therefore largely unknown if all three HLA class I antigens: HLA-A, -B, and -C are co-expressed in class I positive tissues. A few studies demonstrated that both HLA-A and -B are expressed in bone marrow and colon epithelium [17], [22], [28]. Because these studies have used IHC as the primary technique, the comparison between HLA-A and -B loci was at best semi-quantitative and an absolute comparison was not possible.

There is evidence that the HLA-A locus is regulated separately from the -B locus in some tissues. Recently, we showed that cell surface expression of HLA-B is low or absent on human mesenchymal stem cells (hMSC) while HLA-A is fully expressed [29]. While it is common to see locus or allele-specific down regulation in tumor cells, this was the first report in normal human cells. Such divergence of classical HLA class I expression in stem cells indicates that separate developmental programs may control the expression of classical HLA loci during normal cell differentiation and demonstrates that HLA class I expression should be revisited using locus specific (-A, -B, -C) or even allele-specific reagents.

In this study, we have expanded the scope and studied surface expression of HLA-A and -B alleles on pluripotent embryonic stem cells, multipotent stem cells (hMSC and human hematopoietic stem cells (hHSC)) and some of their end-stage progenies (different subsets of lymphocytes and *in vitro* differentiated adipocytes and osteoblasts).

## Materials and Methods

### Cell Lines and Culture Conditions

**Figure 1:**
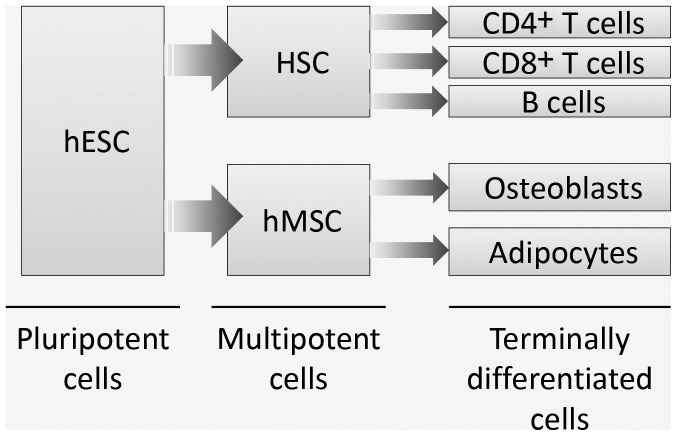
A schematic diagram representing the order of differentiation of the cells used in this study.

In this study two embryonic stem cell lines, four hMSC lines, bone marrow (BM) aspirates (n = 7) and peripheral blood mononuclear cells (PBMC) from healthy volunteers (n = 7, different from the BM donors) were used (Table 1). These cells represent different levels of differentiation as outlined in Figure 1.

**Table 1:** Description of cell lines.

Cell type	Name	Genomic HLA-type			
		A*	A*	B*	B*
hESC	hESC-huES9	03	11	07	51
	hESC-KMEB2	02	66	13	38
hMSC	hMSC-ToB11-5	02	32	07	50
	hMSC-ToB11-10	01	03	08	57
	hMSC-ToB11-13	02	26	07	39
	hMSC-Tert4	02	03	07	27
hHSC and BM T-cells	BM-ToB11-2	02	03	07	40
	BM-ToB11-5§	02	32	07	50
	BM-ToB11-6	02	24	07	44
	BM-ToB11-8	01	26	07	08
	BM-ToB11-10§	01	03	08	57
	BM-ToB11-13§	02	26	07	39
	BM-ToB11-14	01	32	08	27
PBMC	PBMC31	01	03	07	08
	PBMC33	01	02	08	40
	PBMC34	02	11	07	18
	PBMC35	01	03	08	27
	PBMC36	01	26	07	08
	PBMC37	02	23	08	41
	PBMC38	01	02	08	40

§ the BM aspirates also used to establish the hMSC mentioned above.

The derivation, characterization and routine culture of hESC lines (huES9 and KMEB2) has previously been reported [30]–[32]. Cells were cultured on Matrigel (BD Biosciences, San Jose, CA, USA) in mouse embryonic fibroblast-conditioned medium essentially as described in ‘Protocols for Maintenance of Human Embryonic Stem Cells in Feeder Free Conditions’ from Geron Corporation (Menlo Park, CA, USA). Briefly, mouse embryonic fibroblast-conditioned medium was prepared by incubating mitotically inactivated mouse embryonic fibroblasts for 24 hours in medium consisting of Knockout D-MEM, 15% knockout serum replacement, 1% Penicillin-Streptomycin, 1% MEM non-essential amino acids (without glutamine, Cat. No.: 10370-070), 1% GlutaMAX (all from Life Technologies, Taastrup, Denmark), 0.5% human serum albumin (CSL Behring, King of Prussia, PA, USA), and 0.1 mM β-mercaptoethanol (Sigma-Aldrich, St. Louis, MO, USA). This medium was sterile filtered and supplemented with 5–8 ng/ml recombinant human fibroblast growth factor (FGF) (Peprotech, London, UK) immediately before use. Cells were routinely passaged at a 1∶3 ratio using 0.05% trypsin-EDTA (Life Technologies).

hESC were cultured for 3 days after passage before the addition of human recombinant IFNγ (Gibco, Life Technologies). Following incubation for 24, 48 and 72 h periods, respectively, cells were washed in PBS and collected by mechanical scraping in PBE (PBS containing 2 mM EDTA and 0.5% BSA) and stained with monoclonal antibodies for flow cytometry (see antibodies in Table S1).

hMSC and BM mononuclear cells (BMNC) were established from bone marrow aspirates according to a previously established method [29]. Briefly, 20 ml of bone marrow were aspirated into a syringe containing 2 ml of heparin (5000 IU/ml, Amgros, Copenhagen, Denmark), mixed thoroughly and rapidly diluted with 25 ml of complete RPMI (RPMI enriched with 10% fetal bovine serum (FBS), L-glutamine, penicillin and streptomycin, all from Life Technologies) containing 30 IU/ml heparin. The mixture was further diluted with an equivalent amount of PBS without Ca^2+^ and Mg^2+^ (Life Technologies) but containing 30 IU/ml heparin. The diluted marrow aspirate was transferred carefully on to 20 ml of Lymphoprep (Axis-Shield PoC AS, Oslo, Norway) in 50 ml tubes and centrifuged for 40 minutes at 500 × g with the acceleration and brake set to minimum. The interface layer was transferred to new tubes and washed twice with complete RPMI (+heparin 30 IU/ml). The isolated BMNC were used to generate hMSC lines or frozen in 90% heat inactivated FBS and 10% DMSO (Sigma-Aldrich) for later flowcytometric analysis. hMSC were generated by seeding 10^7 ^BMNC in a 75 ml culture flask in 15 ml of complete MEM (MEM enriched with 10% FBS, L-glutamine, penicillin and streptomycin, all from Life Technologies) and incubated at 37°C, 5% CO_2_ for 5 days. The non-adherent cells were removed and the adherent cells were subsequently trypsinized with TryPLE express (Life Technologies) and transferred to new 75 ml culture flasks. hMSC properties were confirmed by flow cytometry (positive for CD73, CD90, CD105, CD146 and negative for CD34, data not shown) and by their ability to differentiate to adipocytes and osteoblasts (see differentiation protocols section).

### Genomic HLA Typing

Genomic DNA was purified from the thawed cells using QiaAmp DNA mini kit (QIAGEN, Hilden, Germany) according to the manufacturer’s protocol. Low resolution HLA typing of the cell lines and donors was performed using LabType SSO (Sequence-specific oligonucleotide probes) (One Lambda, Los Angeles, CA, USA) using a Luminex 100 IS (Luminex Corp., Austin, TX, USA).

### Antibodies

The antibodies used are summarized in Table S1.

### Flow Cytometric Staining

The frozen fraction of the bone marrow aspirate was thawed, washed twice with pre-heated (37°C) fresh medium (complete RPMI), re-suspended in medium and left at room temperature for 30 minutes. The cells were then spun down at 500 × g for 5 min and re-suspended in medium at a concentration of 10^7^ cells/ml.

Adherent cells were incubated with TryPLE express (Life Technologies) for 5–8 minutes until most of the cells were brought into suspension. Then cells were washed twice and re-suspended in medium.

Primary antibody staining with fluorochrome-conjugated antibodies was performed using a maximum of 10^6^ cells in 100 µl of medium with the amount of antibody recommended by the manufacturer. All HLA antibodies were titrated and used in concentrations saturating the staining. We found that final concentrations of 1∶2000 of HLA-A2, 1∶10 of HLA-A3, 1∶500 of HLA-B7, 1∶100 of HLA-B8 and 1∶10 of HLA B-27 antibodies were sufficient. The cells were incubated for 30 minutes (all staining incubations were performed in the dark at 4°C) and washed twice with PBE (PBS containing 2 mM EDTA and 0.5% BSA). Samples stained with mouse anti-human antibodies, not conjugated with a fluorochrome, were subsequently stained with FITC-conjugated goat anti-mouse IgG/IgM antibody and incubated for another 30 min., then washed twice with PBE. If further staining was needed the sample was incubated with mouse serum (Dako, Glostrup, Denmark) for 30 min (to block available binding sites on the secondary antibody) and washed twice and then stained with fluorochrome-conjugated murine antibodies for 30 min. as well. After staining, the cells were re-suspended in PBE containing 1% formaldehyde solution and kept in the dark at 4°C. The cells were analyzed by flow cytometry immediately or at the latest the next day.

hHSC were defined by being positive for CD34 while negative for CD38 and lineage markers (CD3, CD4, CD8, CD14, CD16, CD19, CD20 and CD56) [33].The fluorochrome conjugation of the antibodies is detailed in Table S1. Mature CD4^+^ T cells were defined by gating on the CD4 and CD3 double positive population. Mature CD8^+^ T cells were defined by gating on the CD3 and CD8 double positive population, both for BM and peripheral blood-derived cells.

All flow cytometry was carried out using CyAn ADP from Beckman Coulter and the results were analyzed using the Summit 4.3 program. For direct immunofluorescence, MEF (molecules of equivalent fluorochrome) values were calculated from the MFI (mean fluorescence intensity) and a standard curve made by running FluoroSpheres (Dako) the same day and with the same settings. Samples stained by indirect immunofluorescence, ABC (antibody binding capacity) values were calculated from MFI and a standard curve produced by using the QIFI kit (Dako) beads coated with different known numbers of Ig molecules. The MEF/ABC value of a relevant isotype control staining was subtracted from the corresponding HLA antibody MEF/ABC value, thus calculating the specific MEF/ABC value for each HLA antibody.

### Differentiation of hMSC to Adipocytes and Osteoblasts

Differentiation of established hMSC to adipocytes and osteoblasts was done as published [34] with minor modifications. hMSC were expanded in culture and then seeded in 6 well plates in complete MEM at ∼90–95% confluence. Next day, the medium was replaced with either adipogenic conditioning medium for differentiation to adipocytes consisting of MEM supplemented with 10% horse serum (Sigma-Aldrich), 100 nM dexamethasone (Sigma-Aldrich), 0.25 mM 3-isobutyl-1-methylxanthine (IBMX) (Sigma-Aldrich), 3 µg/ml insulin (Sigma-Aldrich), and 1 µM rosiglitazone (BRL-49653) (kindly provided by Novo Nordisk, Denmark) or osteogenic conditioning medium consisting of MEM supplemented with 10% FBS, 10 nM dexamethasone, 10 nM vitamin D (1,25-dihydroxyvitamin D3) (LeoPhama, Ballerup, Denmark), 10 mM β-glycerophosphate (Calbiochem-Merck, Darmstadt, Germany) and 50 µg/ml vitamin C (L-ascorbic acid) (Wako Chemicals, Osaka, Japan) for differentiation to osteoblasts. Induction medium was changed every third day.

Adipocyte differentiated cells were harvested at day 13 (longer differentiation was not possible because the cells would become too fragile for flow cytometry). Osteoblast differentiated cells were harvested after 16 days of differentiation. In both cases, cells were trypsinized (5–8 minutes at 37°C) and then re-suspended in complete MEM, followed by flowcytometric staining (Table S1).

### Oil Red O Staining for Adipocyte Differentiated Cells

Differentiation of hMSC to adipocytes was confirmed by Oil Red O staining [34]. The cells in the culture dish were washed with PBS, fixed with 4% paraformaldehyde for 10 minutes at room temperature, rinsed with 3% isopropanol and stained with Oil Red O (ORO) (Sigma, Steinheim, Germany) for 1 hour at room temperature and then washed twice with distilled water. The staining solution was prepared by dissolving 25 mg of ORO in a mixture of 5 ml of 100% isopropanol and 3.3 ml of water, incubated for 15 minutes at room temperature and then filtered through Whatman filter paper (Q-Max CA-S, 0.20 µm pore size, Frisenette APS, Knebel, Denmark) before use. The stained cells were examined microscopically for formation of fat droplets inside the cells.

### Alizarin Red Staining for Osteoblast Differentiated Cells

Osteoblast differentiation was confirmed by matrix mineralization visualized by Alizarin Red staining [34]. Briefly, cells in four well plates were washed with PBS, fixed with 70% ice-cold ethanol for 1 hour at −20°C, rinsed with distilled water, stained with 40 mM Alizarin Red (pH 4.2, Sigma-Aldrich, USA) for 10 minutes at room temperature under gentle rotation. Excess dye was removed with H_2_O followed by a brief wash with PBS for 2 minutes under gentle rotation.

### Quantification of Alkaline Phosphatase Activity

Cells were plated in 96 well plates at a density of 6000 cells/well and induced to osteogenic differentiation as described above. On day 7, cells were washed with PBS, rinsed with TBS, fixed with 3.7% formaldehyde for 30 seconds, and then 100 µl of reaction substrate solution (1 mg/ml p-nitrophenylphosphate (Fluka, USA) in 50 mM NaHCO_3_ (pH 9.6) with 1 mM MgCl_2_) were added and incubated for 20 min at 37°C. Finally 50 µl of 3 M NaOH were added to stop the reaction and the absorbance was measured at 405 nm.

### Gene Expression Analysis for Differentiated Cells

During MSC differentiation to both adipocytes and osteoblasts, cells were harvested at day 12 and 16 respectively, mRNA expression of the osteogenic marker, Collagen 1, and the adipogenic marker, PPAR gamma, were verified using RT-PCR.

### Statistics

Statistical analysis was performed using GraphPad Prism 5 software. Student *t*-test or one-way ANOVA were used to test differences of means between two or three groups of data, respectively. P values <0.05 were considered significant and marked with * (P<0.01 marked with ** and values <0.001 with ***). Error bar signifies mean +/− SEM.

### Ethical Approvals

The study was reviewed and approved by the Ethical Committee for the Region of Southern Denmark with the issue No. 2008-00-92. Written informed consent was obtained from the blood and bone marrow donors with respect to sampling and establishment of hMSC cell lines.

## Results

### hESC Constitutively Express Low Amounts of Cell-surface HLA-A but no Detectable HLA-B

**Figure 2:**
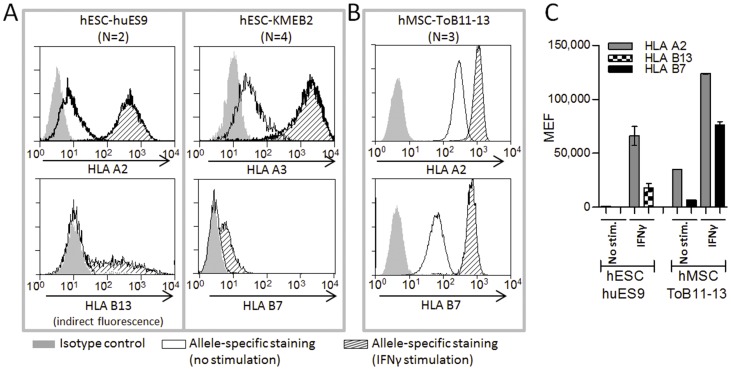
Expression of HLA-A and HLA-B in hESC and hMSC. Representative flow histograms showing HLA-A and -B expression in stem cell lines using allele-specific antibodies (anti-A2, -A3, -B7, and -B13). (A) In separate experiments, hESC lines (huES9 and KMEB2) showed a low expression of HLA-A alleles (A2 and A3, respectively) and no expression of the HLA-B alleles (B13 and B7, respectively). IFNγ stimulation up-regulated HLA-A alleles and induced of modest expression of HLA-B alleles. (B) Panel B shows representative flow histograms of an hMSC cell line (ToB11-13) both un-stimulated and IFNγ stimulated (72 hr) demonstrating a high constitutive expression of HLA-A2 and a relatively low expression of HLA-B7. Both antigens were up-regulated after stimulation with IFNγ. Panel (C) compares the expression of HLA-A and HLA-B between hESC (huES9) and hMSC (ToB11-13) during basal, un-induced conditions and after stimulation for 72 h with 25 ng/µl IFNγ. HLA expression was measured by flowcytometry as molecules of equivalent fluorochromes (MEF).

First, we investigated the basal expression of HLA-A and HLA-B on two well characterized hESC; huES9 [35] and KMEB2 [30] (Figure 2A). These cell lines were chosen because they genetically carry HLA alleles that, if expressed, can be specifically detected by commercial antibodies validated for diagnostic purposes (Table 1). HLA-C expression was not included in the study due to the lack of HLA-C allele-specific antibodies. The allele-specific monoclonal antibody staining showed extremely low, but detectable, HLA-A2 (huES9) and -A3 (KMEB2) expression while HLA-B7 and -B13, respectively, were undetectable on the surface of the cell lines (Figure 2A). However, after stimulation of huES9 with IFNγ (25 ng/ml) for 72 hours, HLA-A2 expression was up-regulated 88 fold (from MEF = 746 to 66,130, mean of triplicates) while HLA-B13 was induced (mean MEF = 18,130, Figure 2C). Similarly, a dramatic up-regulation of HLA-A3 was seen in hESC KMEB2, while only a modest induction of HLA-B7 was seen (Figure 2A). Stimulation for 24 and 48 hours showed less pronounced up-regulation but otherwise similar expression patterns (data not shown).

### hMSC Express High Levels of HLA-A Alleles and Low Levels of HLA-B

hMSC are more differentiated than pluripotent hESC yet still multipotent (Figure 1). We have previously shown that hMSC of bone marrow or adipose tissue origin constitutively express HLA-A alleles on the cell surface, but only weakly HLA-B alleles [29]. In this study, we confirmed those findings in a primary hMSC line (ToB11-13) (Table 1), which showed a relatively high HLA-A2 surface expression (mean MEF = 35,012) and a five times lower but significant HLA-B7 expression (mean MEF = 6,324) under basal growth conditions (Figure 2B and C). The gap between the level of HLA-A2 and -B7 expression was narrowed after stimulation with IFNγ with high levels of both HLA-A2 and -B7 (mean MEF = 123,766 and 76,130, respectively) (Figure 2).

### The Constitutive HLA-A Expression on hMSC is Reduced During Differentiation into Osteoblasts

**Figure 3:**
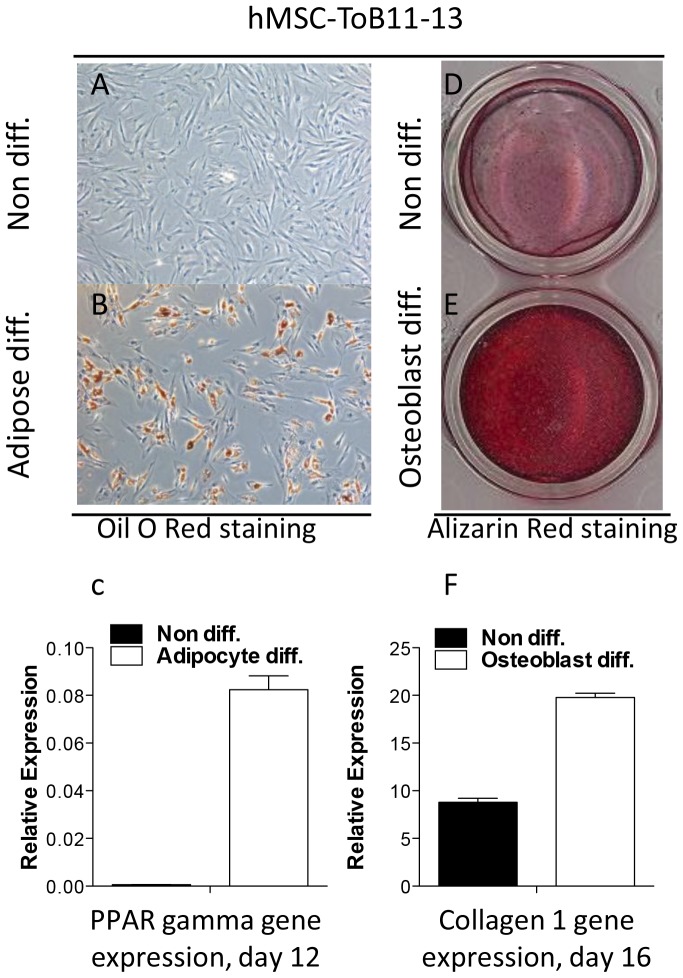
Differentiation of hMSC to adipocytes and osteoblasts. Representative images for Oil O Red and Alizarin red staining demonstrating adipocyte and osteoblasts differentiation of hMSC cell lines cultured under adipogenic and osteogenic conditions, respectively. Non-differentiated cells were not stained (A and D), while the adipocyte differentiated cells were stained with Oil O red (B) and Alizarin red stained the matrix formed by osteoblasts (E). qPCR data shows marked up-regulation of PPAR gamma gene expression after adipocyte differentiation (C) and up-regulation of Collagen 1 gene expression in osteoblast differentiated cells (F).

**Figure 4:**
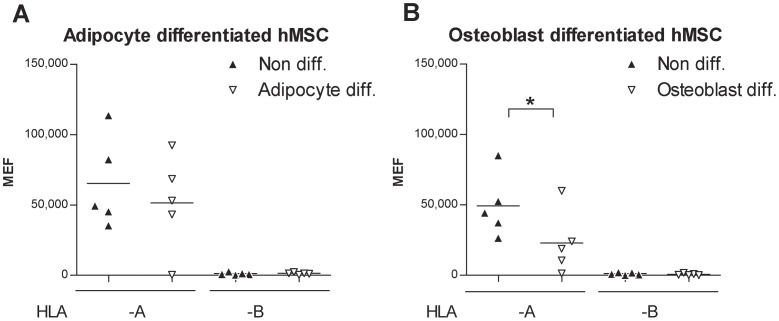
Expression of HLA-A and HLA-B on hMSC and in their differentiated adipocyte and osteoblast progenies. Locus-specific HLA antigen expression before and after 13 days of differentiation of hMSC to adipocytes (A), or 16 days of differentiation to osteoblasts (B) in one hMSC-Tert4 cell line and three primary hMSC cell lines (Table 1). The analyzed HLA-A alleles were HLA-A2 (N = 3) and -A3 (N = 2). The analyzed HLA-B alleles were HLA-B7 (N = 3), -B27 (N = 1) and -B8 (N = 1) (data pooled according to locus, each data point represents mean value for 3 experiments for each cell line).

We next investigated whether the expression of HLA-A and -B could be altered by differentiation from hMSC (multipotent) towards their differentiated adipocyte or osteoblast progenies (Figure 1). Established differentiation protocols were used to drive hMSC into either adipocyte or osteoblast differentiation and successful differentiation was confirmed by expression of cytoplasmic lipid droplets and increased expression of PPARγ mRNA in adipocytes and by extracellular matrix deposition as well as increased expression of Collagen 1 mRNA in osteoblasts (Figure 3C and 3F). Figure 4B shows that HLA-A2 surface expression was significantly reduced during differentiation of hMSC into osteoblasts (P = 0.017). A similar trend was noted during differentiation of hMSC to adipocytes (albeit not significant, P = 0.2) (Figure 4A). Some individual cell lines even exhibited complete absence of HLA-A2 after culture in both differentiation protocols. HLA-B expression remained undetectable in both hMSC and their differentiated progenies (Figure 4).

### hHSC Express High Levels of HLA-A and -B

**Figure 5:**
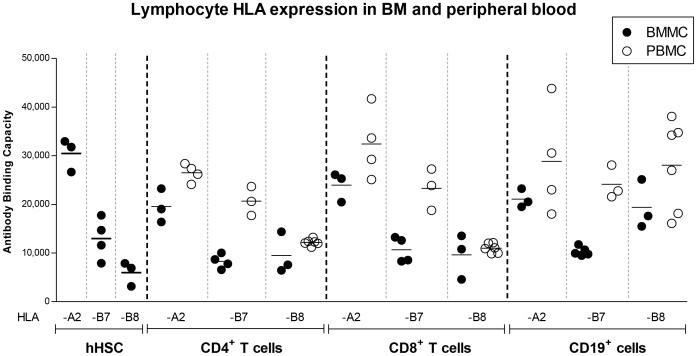
Cell surface HLA expression of hHSC and lymphocytes derived from BM and peripheral blood. All allelic forms of HLA studied were highly expressed in all the cell types shown. However, HLA-A2 was expressed at significantly higher levels than HLA-B7 and -B8 on hHSC, BM CD4^+^ T cells, and BM CD8^+^ T cells. When BM lymphocytes and peripheral blood lymphocytes were compared, there was a marginal (but non-significant) difference in the expression of HLA-A2 and -B8 alleles which tended to have higher expression in peripheral blood than in BM. A significantly higher expression level of HLA-B7 was observed on peripheral blood CD4^+^ and CD8^+^ T lymphocytes. In peripheral blood, the expression of HLA-B8 was significantly lower than HLA-A2 and -B7 on CD4^+^ and CD8^+^ T lymphocytes. Peripheral CD19^+^ lymphocytes expressed similar amounts of HLA-A2, -B7 and -B8.

Like hMSC, hHSC are multipotent stem cells found in BM (Figure 1). hHSC are progenitors to almost all cells in the blood and the immune system. In this study, hHSC where defined by being CD34^+^, CD38^−^ and Lin^-^ where Lin denotes a mixture of lineage markers (CD3, CD4, CD8, CD14, CD16, CD19, CD20 and CD56). BM aspirates were studied using indirect staining with calibration beads allowing quantification of the antibody binding capacity (ABC) (Figure 5).

hHSC expressed high levels of HLA-A which did not differ much (though statistically significant) from that of mature CD4^+^ and CD8^+^ T cells also present in the marrow aspirate (1.8 fold higher in hHSC, P = 0.007 and 1.5 fold higher, P = 0.008, respectively). Also HLA-B expression on hHSC was high and indistinguishable from that of bone marrow CD4^+^ and CD8^+^ T cells (P = 0.7 and P = 0.3, respectively). However HLA-A2 was expressed at significantly higher levels than HLA-B7 and -B8 on hHSC (P = 0.0002), BM CD4+ T cells (P = 0.0045), and BM CD8+ T cells (P = 0.002) (Figure 5).

### Mature Lymphocytes Express More HLA on the Surface in the Peripheral Blood than in BM

The finding that BM-derived lymphocytes expressed more HLA-A2 than HLA-B7 as shown above, prompted us to compare them with peripheral blood lymphocytes (Figure 5). Peripheral T lymphocytes tended to express marginally more HLA-A2 and -B8 than in the bone marrow (although not statistically significant, P>0.11, unpaired *t*-test). However HLA-B7 was expressed significantly more on peripheral T cells than on BM-derived T lymphocytes (P = 0.0007 and P = 0.004 for CD4^+^ and CD8^+^ T cells, respectively).

Further, when the different allelic forms of the proteins (HLA-A2, -B7 and -B8) expressed on the selected peripheral blood lymphocytes were compared, HLA-B8 was significantly less expressed than HLA-A2 and -B7 in CD4^+^ and CD8^+^ T cells (as judged by 1 way ANOVA, P = 0.0001 and P = 0.0001, respectively, Figure 5). This marks a noticeable difference between HLA-B7 and –B8 since the first is expressed two-fold higher than the latter (P = 0.0002 for CD4^+^ T cells and P = 0.0002 for CD8^+^ T cells). In contrast, there was no significant difference between the analyzed HLA alleles in CD19^+^ cells. Peripheral blood CD19^+^ B lymphocytes expressed HLA-A2, -B7 and -B8 levels as high as peripheral CD4^+^ and CD8^+^ T lymphocytes (P = 0.7). BM CD19^+^ cells were likely to be a heterogeneous population of pre-B cells, immature and mature B cells and not evaluated further. Taken together, the levels of HLA-B8 expression were comparable between T cells derived from peripheral blood and BM (P>0.14) and even in hHSC (Figure 5). Thus, the surface expression of HLA class I molecules does not only vary between cell types and levels of differentiation, but it is also influenced by the compartment in which the cells reside. Moreover, variation may even be at the level of individual HLA alleles on the same locus.

## Discussion

Our study compares for the first time the simultaneous quantitative expression of HLA-A and -B alleles on undifferentiated pluripotent embryonic stem cells, more differentiated multipotent stem cells and terminally-differentiated lineage-specific cells with different fates in body compartments.

Allele specific HLA cell-surface expression was markedly different between cells at different stages of differentiation and maturation. Pluripotent hESC are known to express low levels of cell-surface HLA class I based on the staining patterns obtained after incubation with a W6/32 pan HLA class I antibody [36]–[39]. Basak et al. have described low constitutive levels of HLA-A2 cell-surface expression on the H9 human embryonic stem cell line [40] while the surface expression of HLA B or Cw have, to the best of our knowledge, not been investigated. Low but detectable expression of HLA-A, -B, and -C mRNA has been reported as judged by quantitative RT-PCR [36], but this does not imply expression on the protein level because many post transcriptional factors are needed for successful expression and some potentially important ones like TAP1, TAP2, LMP2 and LMP7 have been reported missing at the mRNA level in the HS293 embryonic stem cell line [36]. Our results show that classical HLA class I cell surface expression on hESC does not comprise expression of the HLA-B locus, at least not of the alleles studied in the two hESC lines here. However, after stimulation of hESC with IFNγ the expression of HLA-A alleles increased to high levels as we have previously observed it in multipotent hMSC [29], while only a modest induction of HLA-B was seen. This indicates that the antigen-presentation pathway(s) required for generation, transport and expression of HLA molecules on the cell surface are readily inducible in hESC. The mechanism behind the differential constitutive expression of HLA-A, but not -B remains to be elucidated but could relate to different dependencies on the peptide loading complex [41].

hMSC are multipotent cells that are developmentally more differentiated than hESC. As we have recently reported, hMSC do express high levels of HLA-A, but the expression of HLA-B is substantially down-regulated, though detectable in most cases [29]. Both the examined HLA-A and HLA-B alleles retained the ability to become induced upon IFNγ stimulation as reported previously [29]. Furthermore, after hMSC cell lines were subjected to *in vitro* differentiation according to established validated procedures into some of their downstream lineages (adipocytes and osteoblasts), they showed a marginal reduction in the cell-surface expression levels of HLA-A (though only statistically significant for osteoblast differentiation) when compared to their levels in their parental hMSC lines. This is in accordance with a previous report based on HCA2 and HCA10 antibodies [42] that detect multiple loci of HLA class I molecules [43], [44].

hHSC represent another class of multipotent cells that share the local bone marrow microenvironment with the hMSC. hHSC showed a strong HLA-A cell-surface expression comparable to that found on the surface of mature T and B lymphocytes, and comparatively higher than multipotent hMSC of bone marrow origin. This indicates that cells of hematopoietic lineages are programmed to express high levels of classical HLA class I proteins very early during hematopoiesis. Indeed, a recent study demonstrated that hemangioblasts, a precursor of hHSC and endothelial cells, show an increase in HLA-A2 expression compared with hESC, and this expression increased dramatically as cells differentiated into hHSC [40] in accordance with our results. Interestingly, we find that HLA-B alleles are also relatively strongly expressed on hHSC though still lower expressed than HLA-A. Thus, it is evident that hHSC express three times more of the HLA-A alleles compared to HLA-B alleles.

Greene JM et al. [45] recently studied the relative expression of HLA transcripts in blood leukocytes by pyrosequencing and found that whereas the class I loci contributed differently, an almost equal contribution was found from alleles on the same locus and this contribution varied very little among lymphocyte subsets. Though not incompatible with this, our protein expression data is somewhat in contrast to this, because we found that mature T lymphocytes (CD4^+^ and CD8^+^) exhibited different patterns of expression depending on the type of individual alleles as well as their body compartment of origin. Again, this suggests that post-transcriptional mechanisms are likely to affect HLA class I expression in allele-specific ways. Moreover, the difference in expression between peripheral blood and BM-derived T lymphocytes suggests that HLA surface expression may even be influenced by signals exerted on them by the compartment where they are located even in the absence of overt inflammation or immunization. However, it is surprising that cell surface expression of the HLA-B8 allele remained low in CD4^+^ and CD8^+^ lymphocytes regardless of their origin, while its expression was moderately high in B lymphocytes. This suggests that HLA-B8 allele might be regulated differently from other alleles.

Locus or even allele-specific regulation of classical HLA class I expression may prove important for understanding T cell and NK cell responsiveness in several tissues. The density of expressed peptide-loaded HLA molecules encoded by an individual allele may impact the outcome of an immune response just as their affinity for a given T-cell receptor [46]. This opens for an alternative explanation for the phenomenon that certain HLA alleles are associated with clearing of certain viral infections e.g. HCV [47] and with slow progression of other infections, e.g. HIV [46], [48].

The finding that HLA-A is preponderant in most cells studied could be related to the clinical observation in allogeneic bone marrow transplantation that mismatches to HLA-A is tolerated more poorly than mismatches in HLA-B alleles [49]. However, our finding that HLA-A was down-regulated during differentiation of hMSC toward osteoblasts may be promising for future stem cell therapies using *in vitro* differentiated tissues.

Overall, our findings show that expression of cell-surface HLA-A and -B alleles are regulated individually through different mechanisms in normal human cells, according to the cell type, differentiation state or their location in the body. Future studies should address the specific mechanisms governing allele-specific HLA expression.

## Supporting Information

Table S1 Description of the antibodies used in the study.
(DOCX)
