# Supplementary material for: Separate Developmental Programs for HLA-A and -B Cell Surface Expression during Differentiation from Embryonic Stem Cells to Lymphocytes, Adipocytes and Osteoblasts
Source: PLoS One. 2013 Jan 18;8(1):e54366. doi: 10.1371/journal.pone.0054366 (PMC3548781; doi:10.1371/journal.pone.0054366)
Supplement: Table S1 — Description of the antibodies used in the study. (DOCX) [file pone.0054366.s001.docx]

**Supplementary Table S1: Description of the antibodies used in the study**

| Specificity | Clone | Conjugation | Species/isotype | Producer | Cat. No. |
| --- | --- | --- | --- | --- | --- |
| HLA A2 | BB7.2 | FITC | mouse IgG2b | AbD Serotec | MCA2090F |
| HLA A2 | BB7.2 | Unconjugated | mouse IgG2b | AbD Serotec | MCA2090 |
| HLA A3 | - | Biotin | mouse IgM | One Lambda | BIH0269 |
| HLA B7 | BB7.1 | FITC | mouse IgG1 | AbD Serotec | MCA986F |
| HLA B7 | BB7.1 | Unconjugated | mouse IgG1 | AbD Serotec | MCA986 |
| HLA B8 | - | Biotin | mouse IgG2b | One Lambda | BIH0536A |
| HLA B13 | - | Biotin | mouse IgM | One Lambda | BIH0261 |
| HLA B27 | - | Biotin | mouse IgG2b | One Lambda | B27F50X |
| HLA ABC | G46-2.6 | APC | mouse IgG1 | BD Pharmingen | 555555 |
| CD3 | SK7 | PE | mouse IgG1 | BD Biosciences | 345765 |
| CD3 | BW264/56 | VioBlue | mouse IgG2a | Miltenyi Biotec | 130-094-363 |
| CD4 | RPA-T4 | PE | mouse IgG1 | BD Pharmingen | 555347 |
| CD4 | SK3 | PerCP | mouse IgG1 | BD Biosciences | 345770 |
| CD8 | DK25 | PE | mouse IgG1 | Dako | R0806 |
| CD14 | M5E2 | PE | mouse IgG2a | BD Pharmingen | 557154 |
| CD16 | B73.1 | PE | mouse IgG1 | BD Biosciences | 332779 |
| CD19 | HIB19 | PE | mouse IgG1 | BD Pharmingen | 555413 |
| CD19 | HD37 | APC | mouse IgG1 | Dako | C7224 |
| CD34 | 518 | APC | mouse IgG1 | BD Pharmingen | 555824 |
| CD38 | HIT2 | PerCP Cy5.5 | mouse IgG1 | BD Pharmingen | 551400 |
| CD44 | G44-26 | FITC | mouse IgG2b | BD Pharmingen | 555478 |
| CD44 | 515 | PE | mouse IgG1 | BD Pharmingen | 550989 |
| CD146 | MUC18 | Unconjugated | mouse IgG1 | AbD Serotec | MCA2141 |
| CD45 | HI30 | FITC | mouse IgG1 | BD Pharmingen | 555482 |
| CD45 | T29/33 | PE | mouse IgG1 | Dako | R7087 |
| CD45 | 2D1 | PerCP | mouse IgG1 | BD Biosciences | 345809 |
| CD45 | HI30 | Pacific Blue | mouse IgG1 | BioLegend | 304029 |
| CD56 | MY31 | PE | mouse IgG1 | BD Biosciences | 345810 |
| Mouse Ig | Polyclonal | FITC | Goat Ig | BD Pharmingen | 555988 |
